# ‘Skullduggery’: Lions Align and Their Mandibles Rock!

**DOI:** 10.1371/journal.pone.0135144

**Published:** 2015-11-04

**Authors:** Vivienne L. Williams, Andrew J. Loveridge, David J. Newton, David W. Macdonald

**Affiliations:** 1 School of Animal, Plant & Environmental Sciences, University of the Witwatersrand, Johannesburg, South Africa; 2 Wildlife Conservation Research Unit, Department of Zoology, University of Oxford, Oxford, United Kingdom; 3 TRAFFIC, East/Southern Africa Regional Office, Pretoria, South Africa; University of Utah, UNITED STATES

## Abstract

South Africa has legally exported substantial quantities of lion bones to Southeast Asia and China since 2008, apparently as part of the multinational trade substituting bones and body parts of other large cats for those of the tiger in wine and other health tonics. The legal sale of lion bones may mask an illegal trade, the size of which is only partially known. An observed component of the illegal trade is that quantities of skeletons are sometimes declared falsely/fraudulently on CITES export permits. Furthermore, there are emerging concerns that bones from tigers reared in captivity in South Africa and elsewhere are being laundered as lion bones using CITES Appendix II permits. There is therefore a need for tools to monitor the trade in lion body parts and to distinguish between lions and tigers. Our research indicates that it is possible to use skeletons, skulls and cranial sutures to detect misdeclarations in the lion bone trade. It is also possible to use the average mass of a lion skeleton to corroborate the numbers of skeletons declared on CITES permits, relative to the weight of the consolidated consignments stated on the air waybills. When the mass of consolidated consignments of skeletons destined for export was regressed against the number of skeletons in that consignment, there was a strong correlation between the variables (r^2^ = 0.992) that can be used as a predictor of the accuracy of a declaration on a CITES permit. Additionally, the skulls of lions and tigers differ: two cranial sutures of lions align and their mandibles rock when placed on a flat surface, whereas the cranial sutures of tigers are not aligned and their mandibles rest naturally on two contact points. These two morphological differences between the skulls of tigers and lions are easy to observe at a glance and provide a method for distinguishing between the species if illegal trade in the bones is suspected and the skulls are present. These identifications should ideally be confirmed by a DNA test to provide rigorous evidence to prosecute offenders violating CITES regulations.

## Introduction

Tiger (*Panthera tigris* L.) parts are one of the most lucrative animal parts sold on the illegal wildlife market [1]. In the mid-1990s, as tiger populations declined across Asia in response to persecution and illegal trade, it was noted that the body parts of large Asian felids such as leopards were being used as tiger substitutes in manufactured ‘tiger’ products with increasing prevalence and that images of lions were appearing on the labels of manufactured Chinese medicines [2–5]. It wasn’t until 2005, however, that lion was confirmed as an ingredient in the ‘bone strengthening wine’ manufactured by a company in Guilin, China [6]. At the time there was no evidence that bones from wild lions were being used in the production of the wine and the carcasses were reportedly sourced from a nearby captive breeding facility [6]. By 2008, however, bones from African Lions (*Panthera leo* L.) were being exported from Africa to Asia [7].

**Fig 1:**
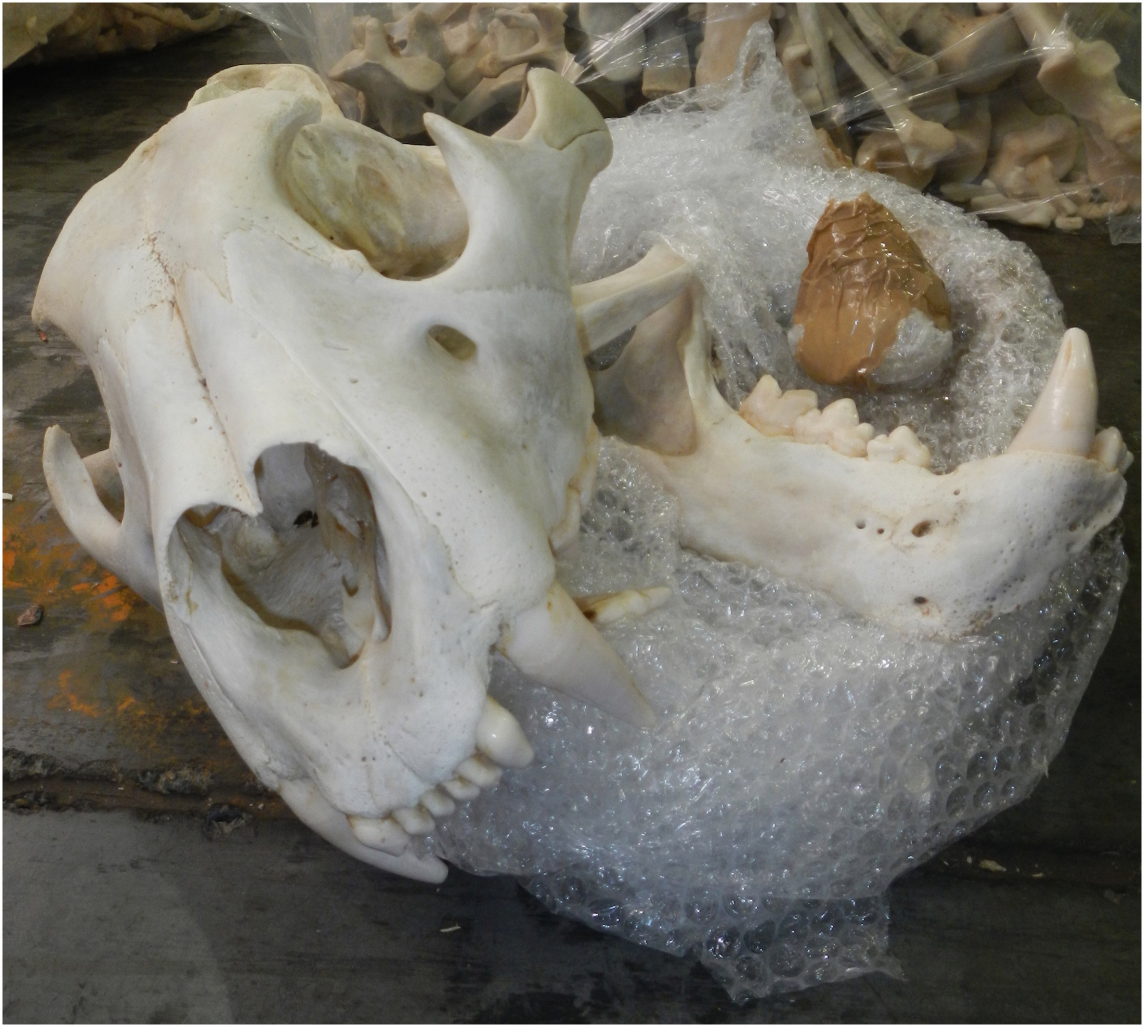
Skull of an African Lion. [Photo: V.L. Williams].

South Africa has exported substantial quantities of lion bones (>1160 skeletons from 2008–2011) to Southeast Asia and China since 2008 as part of the multinational trade in tiger substitutes to meet the demand for ‘tiger’ bone remedies [7] (Fig 1). The legal sale of lion bones may mask an illegal trade, the size of which is only partially known. An observed component of the illegal trade is that quantities of skeletons are sometimes declared fraudulently on CITES export permits. False information provided by an exporter is generally difficult to detect and only customs officials and the police can conduct random cargo inspections to examine the contents of consignments and thereby identify irregularities and potentially fraudulent trade. One way of detecting permit irregularities of this nature, however, would be to compare declared skeletal weights with the expected mass of lion skeletons—but beyond a few inconsistent ‘guestimates’, this information is unavailable (although estimates for tigers of 6–12 kg of bone per skeleton were reported by Nowell & Jackson [8] and Nowell [4], 10–12 kg by Moyle [9], and 20 kg by Gratwicke *et al*. [10]). The availability of this information could be used as a convenient method to facilitate the remote corroboration of the quantities of skeletons declared on CITES permits, relative to the weight of the consolidated consignments stated on the air waybills, without physically having to inspect the cargo unless further permit violations are suspected. However, published studies on aspects of the anatomy and morphology of large felids have concentrated mainly on body proportions, adaptive differences, and functional implications [11] and not skeletal mass.

While lion bones are being substituted for tiger bones in tonics, there is also an emerging concern that bones from tigers reared in captivity in South Africa and elsewhere are being laundered as lion bones using CITES Appendix II permits or are not being declared at all [7]. Information provided by the Free State Province (South Africa) permit Issuing Authority, for example, confirms the need to scrutinize and monitor the national transport and trade of tiger carcasses and skins (Werner Boing, pers. comm. 12/03/2015), and the motives thereof. Prior to 2012, no applications had been made to transport skins and carcasses from euthanized tigers from one location to another within the Free State Province. In 2012, however, a permit to transport one Bengal Tiger skin was granted; in 2013, permits to transport two Bengal Tiger carcasses were granted; and in 2014, applications to transport 22 Bengal Tiger carcasses were granted (Werner Boing, pers. comm. 12/03/2015). Furthermore, permits issued to export the ‘flat skins’ of Bengal Tigers from the Free State to the Province of KwaZulu-Natal amounted to five in 2013 and 17 in 2014. Since the KwaZulu-Natal coastline has two of the busiest bulk ports in South Africa (Durban and Richards Bay), a question arises as to whether these seaports are being used as exit points for illicit cargo. The destination of the carcasses is unknown.

Limitations in the South African legislation applying to endangered exotic animals have fostered this allegedly growing and unregulated domestic trade in tigers. A challenge to authorities monitoring and policing both the legal and illicit wildlife trade, however, is that, in the absence of DNA testing to confirm taxonomic identity, it is nearly impossible, without expert knowledge, to distinguish between lion and tiger bones [12]. Species identification is paramount to the enforcement of CITES regulations [13], therefore there is also a need for practical methods that can be used by customs officials to distinguish between lion and tiger bones with near certainty when the species identity is in question.

In light of this need for a new set of tools to cope with the now established trade in lion bones and concerns that tiger bones are being laundered in South Africa, the aim of this paper is to provide a guide to the average mass of lion skeletons and skulls, and a means for distinguishing the skulls of lions from tigers. These needs arose as a result of our study on the trade in lion bones from South Africa to East-Southeast Asia [7], and the challenges that emerged in interpreting (i) the quantities of skeletons declared on the permits, (ii) information on the mass of skull-less skeletons in the absence of data on skull mass, and (iii) whether a bag of clean felid bones weighing 13kg was that of a large lion or tiger, and whether other smaller tigers were part of that consignment (this latter need was not resolved). Since these methods were ultimately beneficial to our study, at the very least these tools will also assist officials to detect anomalies in one aspect of the large felid bone trade and the deliberate falsification of information on CITES permits.

## Methods

Seventy-one wild-origin African Lion skulls with the crania and mandibles were weighed (34 ♀, 23 ♂, 14 sex unknown) from the Ditsong Museum of Natural History, Pretoria (DMNH), the Oxford University Museum of Natural History (OUMNH), the School of Animal, Plant & Environmental Sciences (WITS) and Evolutionary Studies Institute (EIS) at the University of the Witwatersrand, Johannesburg, and the Zimbabwe Parks & Wildlife Management Authority (ZimParks), Hwange National Park, Zimbabwe. Specimen accession numbers, skull mass, country of origin, sex and mortality dates are listed in S1 Table. Specimens from Hwange are not formally curated by ZimParks, but were given study identification numbers by the Hwange Lion Project of WildCRU (Oxford University). The specimens excluded skulls from known juveniles and sub-adults.

The post-mortem ages of the lion specimens at DMNH ranged from 40–120 years old (although newer but undated skulls are in the collection) and included 17 skulls from South Africa, eight from Namibia, three from Sudan, one each from Malawi, Mozambique and Zimbabwe, and seven skulls from unknown localities (S1 Table). The most-mortem ages of the lion specimens from OUMNH ranged from 68 to >155 years old, and included 12 skulls from Sudan, three from South Africa, two from Uganda, one from Tanzania, and 6 skulls of unknown origin (S1 Table). One specimen from India in the collection is presumed to be an Asiatic Lion (*Panthera leo* subsp. *persica*). Post-mortem ages of the skulls from WITS and EIS are mostly unknown, and those from Hwange ranged from 1.5–4.5 years.

Skeletal mass was obtained for 546 lions of varying sex, age, size and ‘completeness’ (i.e. the presence or absence of the skull) (Parts F,G,H in S1 Table). Of the 546 skeletons, 36 skeletons were individually weighed: (1) two adult males, one with and one without a skull from DMNH (AZ2389 and AZ565 respectively) (post-mortem ages unknown), and (2) 34 bags of skeletons reported to be *en route* to Southeast Asia from South Africa in 2013 (10 skeletons with skulls; 10 skeletons without skulls; 14 skeletons where the mass had been noted but not the presence/absence of skulls). In addition, the mean mass for 510 skeletons consolidated into 15 consignments exported to Southeast Asia from South Africa from 2009 to 2014 was obtained; the information for these skeletons was made available through South African government agencies of actual exports where both the mass and the number of skeletons per consignment was recorded. The data retrieved for these 510 skeletons were: (i) linearly regressed to show the correlation between the variables, and (ii) compared with the mass of the 36 weighed skeletons, to detect consignments where the mean mass per skeleton deviated from the mean mass of the individually weighed skeletons and were therefore potentially anomalous permit declarations. While the post-mortem ages of the exported skeletons was not known, they are presumed to have been mostly harvested from carcasses within several months to one year of the lions being killed. This presumption is partly based on photographs of bagged skeletons where oily residues and some soft tissue were still evident on the bones and also limited information on when some of the lions were hunted before their bones were exported. While the samples excluded cubs, the presence of sub-adults cannot be ruled out.

While lions and tigers are osteologically very similar, they are not identical and a *“plethora”* of distinguishing characters has been proposed in the past that were often *“semantically vague resulting in interpretive confusion”* [14]. For the purposes of use by customs officials, however, identifying character traits should ideally be user-friendly, easily distinguished at a glance, should not require equipment and complex measurements of character length and ratios, and also be semantically accessible to a person with no expertise in comparative anatomy. An extensive literature and Internet search was undertaken in this regard, and only seven instructive sources of information on cranial and mandibular differences were found, namely papers by Blanford (1888) [15], Pocock (1929, 1930, 1939) [16–18], Merriam & Stock (1932) [19] and Christiansen (2008) [14], and an Internet blog posted by Raptor’s Nest (2008) [20]. These authors proposed several explicit singular characters for distinguishing between the skulls, but ultimately only two characters met our requirement of being a practical identification tool with an acceptable degree of certainty, namely: (1) the position and alignment of the posterior projections of the nasal-frontal and maxilla-frontal sutures on the cranium, and (2) the ventral profile of the horizontal ramus of the mandible, including the number of contact points the mandibles/jaws have on a flat surface. In addition, three other identifying criteria that provide less certain species determinations are discussed.

To compare cranial and mandibular morphology, 104 specimens were examined from: (1) 82 African Lions (38 ♀, 28 ♂, 16 sex unknown) from DMNH, OUMNH, WITS, EIS and Hwange (S1 Table), and (2) 22 tigers (7 ♀, 11 ♂, 4 sex unknown) from DMNH and OUMNH (S2 Table). The subspecies of tiger were not recorded with the specimens in the museum databases, but origin records accompanying some skulls indicates the likely presence of *Panthera tigris tigris* (Bengal tiger), *P*. *t*. *jacksoni* (Malayan tiger), and at least one subspecies from China (Indochinese, Amur or South China tiger). One ‘Liger’ (*P*. *leo* ♂ x *P*. *tigris* ♀) specimen from National Museum of Bloemfontein (NMB 934), South Africa, was also examined (S4 Fig). The specimens included skulls from juveniles and sub-adults. Photographs of some specimens were taken, usually using a fixed camera-to-subject distance and angle was maintained to exclude comparative errors resulting from different camera settings and method inconsistencies.

Despite an extensive literature search, only one source of information was retrieved on post-cranial skeletal species differences that might assist with identifying bones in the absence of skulls in intercepted cargo. Merriam & Stock [19] described subtle variations in the profiles of the scapula, humerus, radius, femur, and tibia of lions and tigers. However, since we did not have access to complete tiger skeletons we could not make a comparative study of these post-cranial differences and this remains an area for further investigation.

## Results and Discussion

### Skull and Skeleton Mass

The mean skull mass for wild-origin African Lions was 1.3 ± 0.4kg, with skulls ranging in weight from 0.7kg for a lioness specimen collected in South Africa in 1906 (DNHM 385), to 2.7kg for a 7.5 year old male that died in Hwange in May 2013 (Table 1; S1 Table). Known female skulls weighed less than 2.0kg, whereas male skulls will be heavier. Skull mass can be used as a proxy for lion size and age, and heavier skulls from individuals of unknown sex and age are inferred to have originated from bigger lions and, typically, older males. Data from seven known-age lions in Hwange (Part E in S1 Table), and ages estimated for the OUMNH specimens, showed that skull mass generally increased with lion age.

**Table 1:** Mean mass of wild-origin African Lion skulls (cranium and mandible) for individuals of varying sex, size and age.

	Mean skull mass (kg)	± Std. Dev.	n	Range (kg)
Male	1.7	± 0.4	23	0.8–2.7
Female	1.1	± 0.3	34	0.7–1.9
Unknown sex	1.2	± 0.5	14	0.7–2.2
**Total skulls**	**1.5**	**± 0.4**	**71**	**0.7–2.7**

Thirty-eight specimens are from DNHM, 19 from OUNHM, three each from WITS and EIS, and seven from Hwange National Park, Zimbabwe (S1 Table). The samples exclude known juveniles and sub-adults.

While little information could be found on bone desiccation rates and the difference between wet and dry bone mass, bones obtained from recent mortalities with more marrow and soft tissue are expected to weigh more relative to their size when compared to similarly sized older and drier specimens. However, in an experiment on pig bones, Raja *et al*. [21] concluded that most of the wet mass is lost within two years of mortality and thereafter the mass loss approaches an asymptote and there are no significant declines in bone mass with increasing post-mortem age. Assuming a similar principle applies to lion skulls and given that the post-mortem age of nearly all the samples (n = 70; 99%) was greater than two years, the date of specimen collection was not believed to influence the overall comparability of the specimens used in the analyses to calculate the mean skull mass. And, while the heaviest male skull specimens were from recent mortalities in Hwange, the heaviest female skull was collected from a lioness in Namibia in 1964 (DNHM, TM38520; weight = 1.9kg) and the mass of the fresher Hwange lioness skulls (≈6.9 years old) were on average 374g more than the mean skull mass for females.

The average mass of a cleaned lion skeleton without soft tissue was calculated to be 9.0 ± 1.8kg and range from 6.0–13.0kg (Table 2. *Note*, *that the 13kg skeleton could not be ruled out as being from that of a tiger and so we cannot be sure whether 13kg represents a reasonable upper limit for the range*). These skeletons are presumed to be mostly of captive origin because 544 of the 546 skeletons were being exported (see below). The sample of 11 skull-less skeletons weighed on average 2.2kg less than the sample of 11 skeletons known to include the skull (Table 2)–but the upper range for the weight of a male lion skull added to the mass of the skull-less skeleton puts the mean weight in the same range as skeletons with skulls.

**Table 2:** Mean mass of African Lion skeletons for individuals of varying sex, size, age and skeleton completeness (presence/absence of a skull).

Completeness of skeleton	Mean skeleton mass (kg)	± Std. Dev.	n	Range (kg)
Without skull	7.0	± 0.9	11	6.0–9.0 ^a^
With skull	9.2	± 2.3	11	6.5–13.0 ^b^ ^,^ ^c^
Skull presence/absence not recorded ^d^	9.8	± 1.4	14	6.7–11.4
Consolidated consignments (skeletal completeness varies)	9.6	± 1.0	510 ^e^	7.6–11.2
**Total skeletons**	**9.0**	**± 1.8**	**546**	**6.0–13.0**

While samples exclude cubs, the presence of sub-adults cannot be ruled out. Since bones for the trade are mostly obtained from lions following a trophy hunt, the skeletons are presumed to be of mostly captive-origin.

^a^ Range includes a control specimen from DNHM weighing 6.3kg.

^b^ Range includes a control specimen from DNHM weighing 10.7kg

^c^ This specimen could not be ruled as being from a 13kg tiger.

^d^ Individual bags of skeletons were weighed, but the presence/absence of skulls was not noted.

^e^ The 510 skeletons were consolidated into 15 consignments (mean = 34 skeletons per consignment) totalling 4897kg.

The overall mean mass per skeleton was obtained from the mean mass per skeleton per consignment. Consignments were not inspected and hence skeleton completeness is unknown.

The weight of a lion skeleton largely depends on its completeness (particularly the presence of the cranium and/or mandible), size and sex (skeletons from male lions weigh more). Since 99.6% of the skeleton records were obtained from summarized CITES export permit data, we were unable to calculate sex- and age-related skeleton mass because the requisite data are not required on the permits. However, since skeletons exported to East-Southeast Asia from South Africa are frequently sourced from the carcasses of captive-bred animals following a trophy hunt (where normally the skull and skin are taken as a trophy by a hunter), many of the lion skeletons are expected to be skull-less [7] and from males (±33% of lions hunted for trophies in South Africa are female [22]). The actual ratio of male/female and trophy/non-trophy hunted animals is, nevertheless, unknown, since skeletons are also allegedly sourced from an unknown proportion of captive lions that died from natural causes or euthanasia. Hence, the assumption made when calculating the average mass of a lion skeleton was that the specimens were mostly captive-bred, of mixed sex, age, size, source, and post-mortem age, and that the estimates took into account normal variations in specimen weight in relation to these factors.

**Fig 2:**
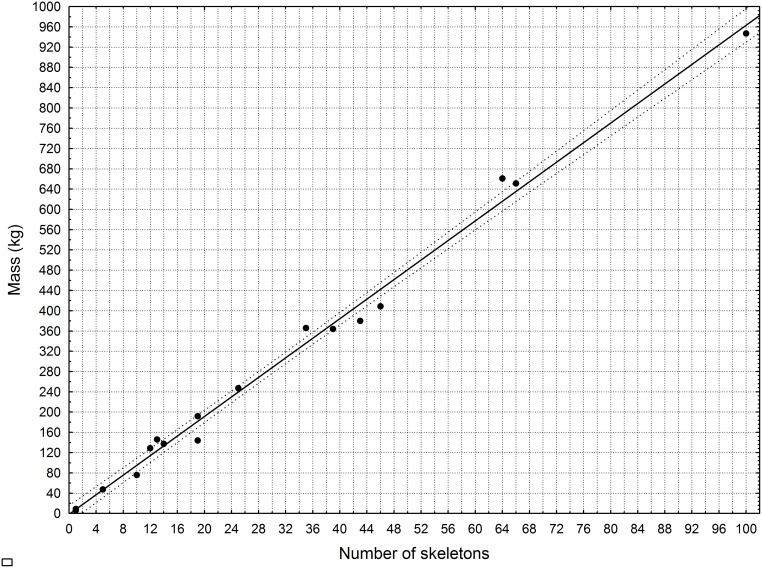
A guide for CITES permit issuers, customs officials and freight forwarding agents on what a consignment of African Lion bones should weigh relative to the number of skeletons declared on the permit or air waybill. The regression was calculated from 510 skeletons consolidated into 15 consignments and two skeletons from DMNH (AZ656; AZ2389). The dashed lines parallel to the regression (solid line) indicate the range within 95% of the mean. The regression equation is y = 9.643x–1.3544, and r^2^ = 0.992. Regression corrected for anomalies (Compare with S1 Fig where anomalies not corrected).

Knowledge on the weight of lion skeletons is indispensable as a tool for (1) detecting anomalies in the mass of a consignment relative to the number of declared skeletons when an exporter/importer applies for a CITES permit, or when an exporter is issued with an air waybill by a freight forwarder to transport the cargo to its declared destination, and (2) estimating how many equivalent skeletons are being exported/imported when only the mass of a consolidated consignment is recorded on a CITES permit or air waybill. Anomalous CITES permit declarations were detected several times during this study; in two instances, the masses of the consignments declared on the permits did not even remotely correspond with what the number of skeletons should have been (S1 Fig) and the average mass of those skeletons was calculated to be 19.8kg each—more than twice the mean mass presented in Table 2. Hence, the cases were interpreted as a deliberate attempt by the exporters to conceal the actual number of skeletons being exported, and the declaration of skeleton numbers on the CITES permits was likely to be fraudulent. When the anomalous data was corrected for (Fig 2) and the number of skeletons declared on these permits was doubled, there was a strong correlation between the variables (r^2^ = 0.992) and the masses of the consignments were within the 95% confidence interval for the expected number of skeletons (Fig 2). This regression can thus be used by law enforcement to detect anomalies.

What are the motives behind the false declarations of skeleton quantities on permits? Unwillingness by exporters to adhere to their legal obligation of providing the correct information when applying for a CITES permit and/or exporting a consignment of skeletons is mainly known to the persons committing the fraud, but their reasons may include: (i) the exporters wish to cover up the extent of this controversial trade and not show the actual numbers of skeletons being freighted to East-Southeast Asia, (ii) the CITES permit they were initially granted does not cover the quantity that is finally packaged for export and they failed to apply for a permit to cover the additional quantity, and (iii) it may be a way of laundering skeletons derived from captive and/or hunted tigers and lions and for which they did not receive an export permit. There is recent evidence to suggest that the trade in lion and tiger bones from South Africa is *“increasing daily and the demand to euthanize* (sic) *tigers has dramatically increased”* (Werner Boing, pers. comm. 11/03/2015). Furthermore, information was received that there are investigations underway in the Free State Province where persons actually hunted tigers, but the province does not allow tiger hunting and would never issue a CITES permit for a hunted tiger (Werner Boing, pers. comm. 12/03/2015). There is therefore sufficient evidence to suggest that fraudulently falsifying documentation is one way that exporters are trying to conceal an illegal trade in tigers and lions.

### Distinguishing Between the Skulls of Lions and Tigers

**Fig 3:**
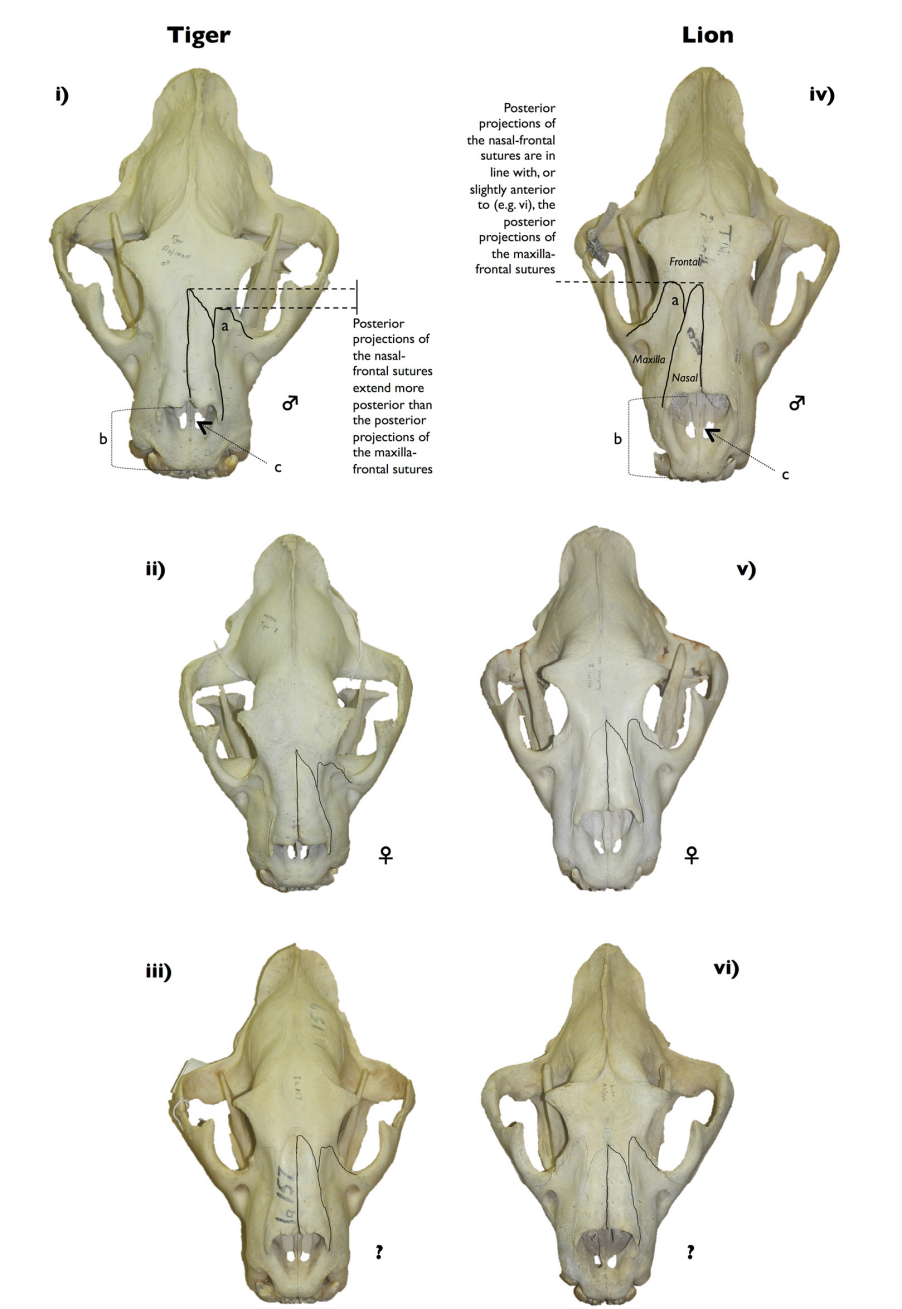
A quick guide to distinguishing between the crania of tigers (i–iii) (AZ1065, AZ772, Ia157) and lions (iv–vi) (TM24004, AZ771, AZ566) using the alignment of the posterior projections of the nasal-frontal and maxilla-frontal sutures. Specimens (i) and (iv) are male, specimens (ii) and (v) are female, whereas specimens (iii) and (vi) are of unknown (?) sex. The cranial sutures have been outlined to show the alignment, and are aligned in lions but not in tigers. Three additional features can also be used with less certainty (indicated by letters a,b,c in i and iv above), namely (a) the angle of the apex of the maxilla-frontal suture, (b) the length of the gap between the premaxillar and the nasals, and (c) the distance between the foramen and the nasals.

One character difference in the crania of lions and tigers is the alignment of the posterior projections (or, apices) of the nasal-frontal suture in relation to the maxilla-frontal suture (Fig 3). In lions, the nasal-frontal suture is in line with, or slightly anterior to, the apex of the maxilla-frontal suture [14,16,18,20] (Fig 3iv–3vi). Occasionally in lions the nasal-frontal suture terminates slightly anterior to the maxilla-frontal suture (e.g. Fig 3vi; Part D in S3 Fig), or very slightly posterior to the maxilla-frontal suture (Table 3; Parts B and C in S3 Fig). In tigers, however, the nasal-frontal suture is either slightly posterior to, or, most often significantly posterior to the apex of the maxilla-frontal suture [14,16,18,20] (Fig 3i and 3ii). The degree to which the nasal-frontal suture is posterior to the maxilla-frontal suture in tigers is variable and subspecies-specific [14,16]. In Bengal tigers (*P*. *t*. *tigris*) for example (the subspecies most commonly kept in captivity in South Africa), the nasal-frontal suture is far posterior to the maxilla-frontal suture [14,16], whereas in Sumatran tigers (*P*. *t*. *sumatrae*) the alignment can be leonine-like with the frontal-nasal suture less posterior to the frontal-maxillary suture [14,16]. We examined no tiger specimens where the sutures were in alignment (Table 3).

**Table 3:** Frequency of maxilla-nasal-frontal cranial suture alignment in lions and tigers.

	*Lions*		*Tigers* ^a^	
Institution Code	Yes	No	Yes	No
**DMNH**	41	0	0	3
**OUMNH**	24	1 ^b^	0	18
**Hwange**	7	1 ^b^	-	-
**WITS**	3	0	-	-
**EIS**	4	1 ^c^	-	-
**Total number of crania**	**79**	**3**	**0**	**21**

^a^ One tiger specimen had no cranium, hence n = 21 and not n = 22

^b^ Nasal-frontal suture terminates slightly posterior to the maxilla-frontal suture

^c^ Nasal-frontal suture terminates slightly anterior to the maxilla-frontal suture

**Fig 4:**
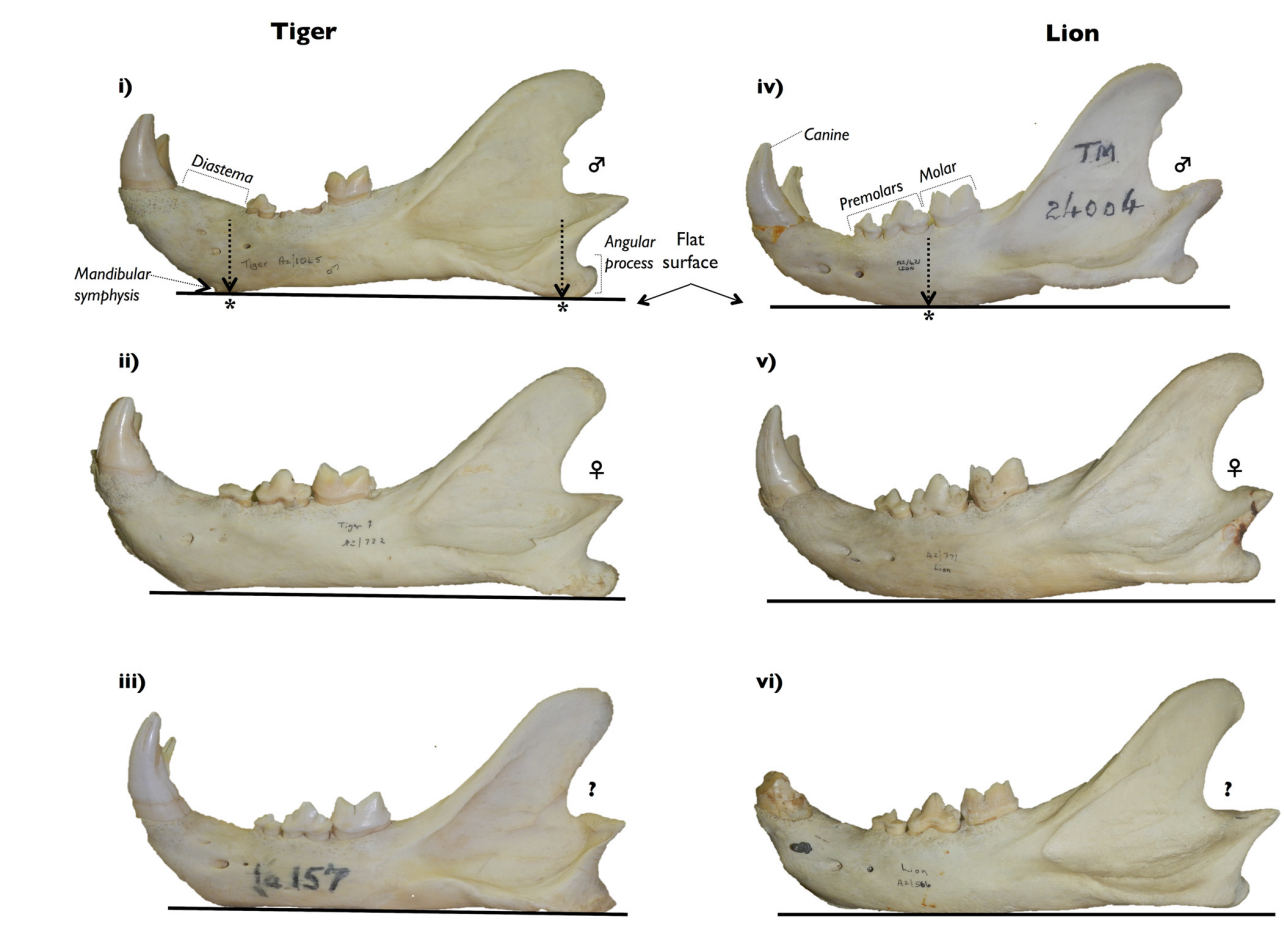
A quick guide to distinguishing between the mandibles of tigers (i–iii) (AZ1065, AZ772, Ia157) and lions (iv–vi) (TM24004, AZ771, AZ566) using the ventral profile of the horizontal ramus. Specimens (i) and (iv) are male, specimens (ii) and (v) are female, whereas specimens (iii) and (vi) are of unknown (?) sex. Tiger mandibles are stable on a flat surface and rest on two contact points, namely the mandibular symphysis below the diastema and on the angular process. Lion mandibles naturally only rest on one contact point on flat surfaces, namely on the ventral margin of the mandible below the region of the molar; hence, due to their convex profile, they tend to rock back and forth. * = contact points of the mandibles on flat surfaces.

A second character that can be used to distinguish between the species is the ventral profile of the mandible, and specifically the shape of the horizontal ramus (Fig 4). In tigers, the mandible naturally rests on two contact points on a flat surface, namely (1) on the mandibular symphysis below the diastema (i.e. the part of the inferior mandible below the gap between the canine and the premolar), and (2) on the angular process (Fig 4i–4iii). In addition, the ventral profile of the mandible is straight or concave [14]. As a result, tiger mandibles always sit firmly when placed on a flat surface [15,17]. In lion mandibles, however, the horizontal ramus mostly has a curved/rounded profile [14] and there is usually only one natural contact point when it is placed on a flat surface, namely the area directly below the second premolar and the molar (the carnassials). As a result, the ventral profile mostly has a convex form (although a few ‘flatish’ ventral profiles were observed where the horizontal ramus was almost straight), and the mandibular symphysis and angular process do not touch a flat surface at the same time. Furthermore, the angular process and the curved area below the carnassial usually do not touch a flat surface at the same time unaided, unless the mandible is unbalanced and rocks backwards. In short, in terms of mandibular stability, ‘lions rock’. Pocock ([17] page 639) emphasized that while the extent to which a lions’ jaw rocks is subject to variation in the degree that it rocks, he had *“never handled a tiger’s skull capable of that movement”*. In our examination of tiger mandibles, however, we found only one tiger mandible that rocked slightly (Table 4; Part F in S2 Fig).

**Table 4:** Frequency of rocking in mandibles of lions and tigers.

	*Lions* ^a^			*Tigers* ^b^	
Institution Code	Yes	No	Intermediate ^c^	Yes	No
**DMNH**	19	3 ^d^	5	0	3
**OUMNH**	24	1 ^e^	0	1 ^f^	17
**Hwange**	6	0	2	-	-
**WITS**	2	1	0	-	-
**EIS**	2	0	2	-	-
**Total number of mandibles**	**53**	**5**	**9**	**1**	**20**

^a^ 15 lion specimens not examined—either because there was no mandible, or the mandible was incomplete, or the mandible was wired to the cranium. Hence, n = 67 and not n = 82

^b^ One tiger specimen had a damaged mandible, hence n = 21 and not n = 22

^c^ Exhibition of this trait was Intermediate, i.e. one half of the mandible (a ‘dentary’) was curved and would have rocked on the contact point below the carnassials, whereas the other dentary had an additional contact point on a flat surface (either the angular process or a bony growth below the mandibular symphysis) that thus prevented the entire mandible from rocking. In some cases in large individuals and/or where front teeth were missing from the specimen, the mandible rocked backwards to rest on the angular process

^d^ One of the three mandibles was prevented from rocking by the presence of a bony growth below the mandibular symphysis (e.g. Part A in S2 Fig)

^e^ Prevented from rocking by the presence of a bony spur below the mandibular symphysis (e.g. Part C in S2 Fig)

^f^ Rocks only slightly (Part F in S2 Fig).

In terms of rocking variations on lion mandibles, we observed nine out of 67 specimens displaying an intermediate form where one half of the mandible (a dentary) had a curved profile and was capable of rocking, whereas the other half had an additional contact point on a flat surface that prevented the entire mandible from rocking (Table 4; S1 Table). The additional contact point on one dentary in these intermediate cases was either the angular process, or the mandibular symphysis. Where the mandibular symphysis was involved, a bony growth below it on one side was sometimes observed (e.g. Part E in S2 Fig). We found no cases in lions, however, of the tiger-like character where both the mandibular symphysis and the angular process rested on a flat surface. We also noted the occasional presence of a small bony spur below the mandibular symphysis on both dentaries that either prevented the entire mandible from rocking (two specimens) or caused the mandible to rock less (S1 Table; Part C in S2 Fig). This bony symphysial spur was noticeable in most, but not all, of the female specimens from Sudan and the one from Tanzania. The ‘Liger’ specimen exhibited lion-like qualities. The mandible rocked and the cranial sutures were in alignment (S4 Fig).

Three other identifying criteria on the crania that are visible in Fig 2 (labelled a, b and c respectively), but which may be less decisive for species determination, are: (a) the angle of the maxilla-frontal suture, (b) the relative distance between the anterior end of the nasals and the start of the premaxillar, and (c) the position of the foramen on the premaxillar relative to the nasals. Firstly, the apex of the maxilla-frontal suture is always truncated in tigers, whereas in lions it tends to be acute (‘a’ in Fig 2i&2iv) [14,20], but it can also be truncated (e.g. the skull in Fig 1). Secondly, the relative distance between the nasals and the premaxillar is typically longer in lions and shorter in tigers (‘b’ in Fig 2i&2iv). And thirdly, the foramen are generally viewable in the middle of the gap between the nasals and the premaxillar in lions, whereas they are closer to the nasals in tigers (‘c’ in Fig 2i&2iv) (with the exception of the tiger cranium in Fig 2iii).

Distinguishing criteria are not limited to the above-mentioned characters of the skull. Indeed, there are other viewable, but less easily distinguishable, comparative differences—especially if a person only has the skull of one species in hand to inspect the attributable traits visually. For example, the interorbital space *“formed by the frontals is wider*, *flatter and even commonly evacuated”* in lions, compared to being *“narrower and always convex”* in tigers [20]. Or, the external opening of the nasal fossae *“is relatively wider; it widens regularly beginning at the lower part”* in lions, whereas in tigers it is *“relatively narrower; it widens regularly*, *only up to a certain height beginning at the lower part”* [20].

If character differences in the elements of the post-cranial skeleton are to be selected for lions and tigers to aid in identification they must, like the cranial differences, be infallible and easy to see at a glance. Ideally these differences should be detectable in bones that are easy to recognise, such as the scapula, pelvis, sacrum, femur, patella and floating bones. Of these bones, Merriam & Stock [19] describe differences in the shape of the glenoid cavity of the scapula (i.e. the cavity on the scapula that articulates with the head of the humerus), and the profile of the femur neck and trochanter. Since we were unable to make a comparative study of these bones, we cannot comment on what features of the post-cranial skeleton, if any, may be appropriate as a species identification guide in the absence of the skull.

Provided consignments of traded bones contain skeletons with skulls, an advantage of using cranial and mandibular morphological characters for provisionally resolving specimen identity is that they can be viewed relatively easily at a glance and do not require complex physical measurements of trait length. However, these identifications must be considered provisional and subject to confirmation by an incontestable DNA test that can be used, if necessary, to prosecute offenders violating CITES regulations. If, however, skeletons are skull-less then we currently know of no reliable means by which to differentiate between the species unless DNA tests are conducted.

## Conclusion

When it comes to skull differences between lions and tigers, the cranial sutures of lions align and their mandibles rock when placed on a flat surface. These morphological differences between the skulls of tigers and lions are a convenient, but not necessarily foolproof, method for distinguishing between the species if illegal trade in the bones is suspected. Our recommendation is that customs officials (especially in South Africa) use these tools when inspecting consignments, or conducting random spot-checks on cargo, destined for East-Southeast Asia as part of an on-going policy to monitor the bone trade and detect irregularities. Furthermore, we recommend that Management Authorities issuing CITES permits adopt the policy that both the number of skeletons and the mass are required on export/import permit applications for consignments, and that they use the regression in Fig 2 to validate the apparent honesty of the declarations. However, a potential weakness that might be exploited by some exporters is that consignments are regularly combined and the consolidated cargo is thus covered by more than one CITES permit. It is not inconceivable that some unscrupulous traders would illegally add more skeletons to the consolidated consignment than is allowed by the CITES permits—so the mass reflected on the air waybill generated by a freight forwarder should also be checked for irregularities a second time against the number of declared skeletons. Since the permit Issuing Authorities cannot check the consignments when they issue the permits, we further recommend that customs officials do spot checks at the port of exit to identify compound shipments by (i) weighing the skeletons, and (ii) checking the number of particularly charismatic and easily identifiable bones—for example, a consignment with more than two femurs or patellas cannot be one specimen.

The morphometric data presented in this paper provide hitherto unavailable information necessary for analysing the trade in lion bones. To this end, these trade tools are useful to CITES Authorities, customs officials, Environmental Management Inspectors (EMIs), and any other interested parties involved in wildlife trade monitoring. Since wildlife crime shows no signs of decreasing [23], these tools will assist with detecting illicit trade and thereby help enforce CITES regulations with respect to two internationally protected species.

## Supporting Information

S1 Fig Linear regression using the same data set as in Fig 2, but anomalous data for two consignments not corrected. (PDF)

S2 Fig A-typical mandibular morphology of lions and tiger. (PDF)

S3 Fig Features of cranial morphology in lions. (PDF)

S4 Fig Liger cranium and mandible. (PDF)

S1 Table Accession numbers and information for African Lion (*Panthera leo*) skull and skeleton specimens: (Part A) skulls from the Ditsong Museum of Natural History (DMNH) (formerly the Transvaal Museum, TM); (Part B) skulls from the Oxford University Museum of Natural History (OUMNH); (Part C) skulls from the School of Animal, Plant & Environmental Sciences (WITS) at the University of the Witwatersrand, South Africa; (Part D) skulls from the Evolutionary Studies Institute (EIS) at the University of the Witwatersrand, South Africa; (Part E) skulls from Hwange National Park, Zimbabwe; (Part F) skeletons from DMNH; (Part G) skeleton specimens from 15 consignments of specimens; (Part H) skeleton specimens from 34 bags of individual skeletons. (PDF)

S2 Table Accession numbers and information for tiger (*Panthera tigris* subsp.) specimens: (Part A) from the Oxford University Natural History Museum (OUNHM); and (Part B) from the Ditsong Museum of Natural History (DMNH). (PDF)
