# Supplementary material for: ‘Skullduggery’: Lions Align and Their Mandibles Rock!
Source: PLoS One. 2015 Nov 4;10(11):e0135144. doi: 10.1371/journal.pone.0135144 (PMC4633142; doi:10.1371/journal.pone.0135144)
Supplement: S1 Fig — (PDF) [file pone.0135144.s001.pdf]

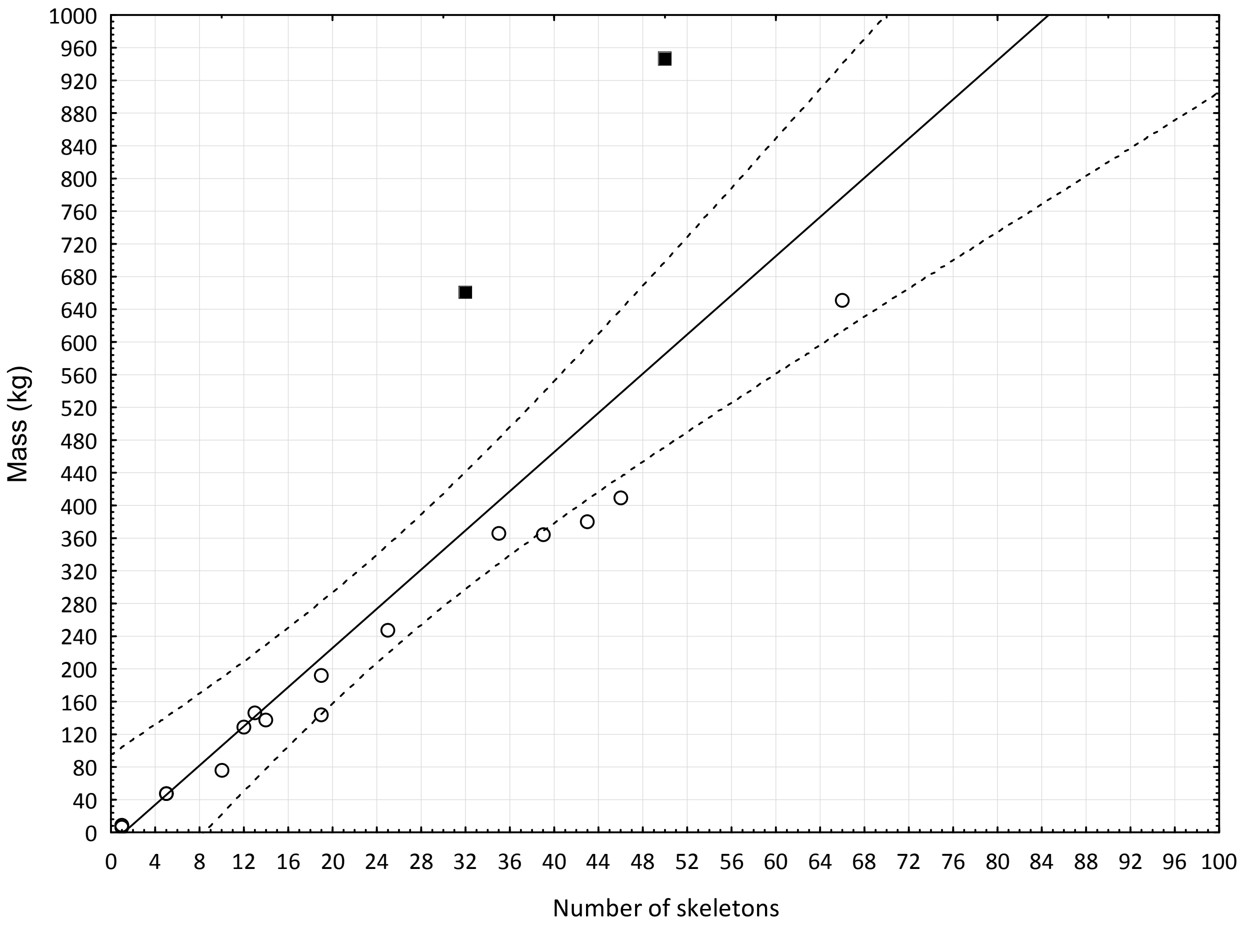

S1 Fig. Linear regression using the same data set as in Fig. 2, but anomalous data for two consignments not corrected. The regression was calculated from 510 skeletons consolidated into 15 consignments, and two skeletons from DMNH (AZ656; AZ2389). The consignments marked as ■ weighed an average of 19.8kg each – which is more than twice the mean mass for skeletons presented in Table 2. The dashed lines parallel to the regression (solid line) indicate the range within 95% of the mean. The regression equation is y=11.983x–14.03, and r^2=0.745. [Compare with Fig. 2 where the anomalies were corrected, and the mean skeleton mass is 9.6kg]
