# Supplementary material for: ‘Skullduggery’: Lions Align and Their Mandibles Rock!
Source: PLoS One. 2015 Nov 4;10(11):e0135144. doi: 10.1371/journal.pone.0135144 (PMC4633142; doi:10.1371/journal.pone.0135144)
Supplement: S2 Fig — (PDF) [file pone.0135144.s002.pdf]

S2 Figures. A-typical mandibular morphology of lions and tiger.

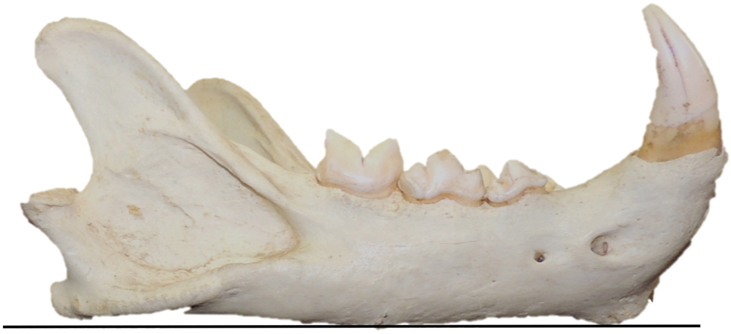

Part A S2 Fig. Non-rocking male lion mandible from South Africa (DMNH, TM38246). Note the bony growth below the mandibular symphysis. *[Photo: V.L. Williams]*

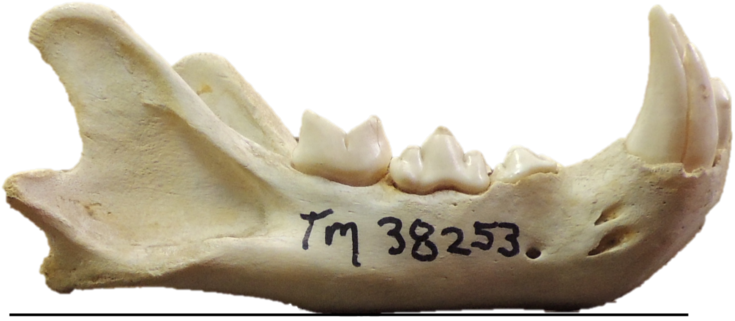

Part B S2 Fig. Non-rocking lioness mandible resting on the mandibular symphysis (DMNH, TM38253). Specimen originates from South Africa. *[Photo: V.L. Williams]*

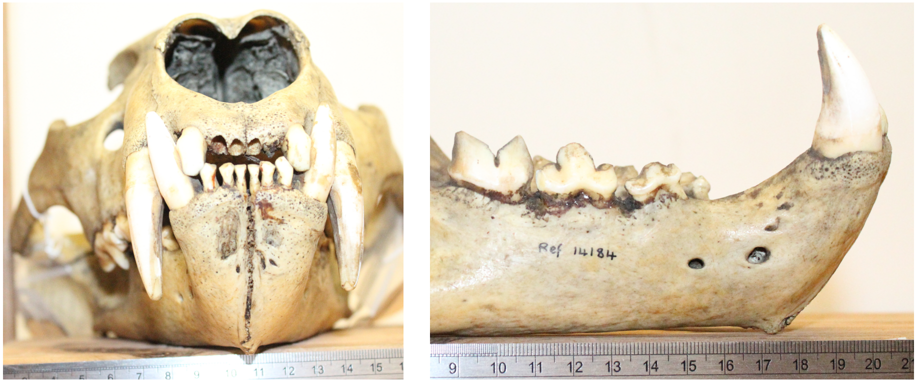

Part C S2 Fig. Non-rocking lioness mandible resting on small, but pronounced, symphysial spur (OUMNH 14184). Specimen is of a 3 to 4 year old female from Sudan. Also note the almost straight ventral profile of the horizontal ramus. There were eight specimens in OUMNH with this type of symphysial spur; four specimens originated from Sudan, one from Tanzania, one from India, and two were of unknown origin. Seven of these specimens were female. These spurs were noticeable on most, but not all, of the 12 specimens from Sudan in total and also the one from Tanzania. *[Photo: A.J. Loveridge]*

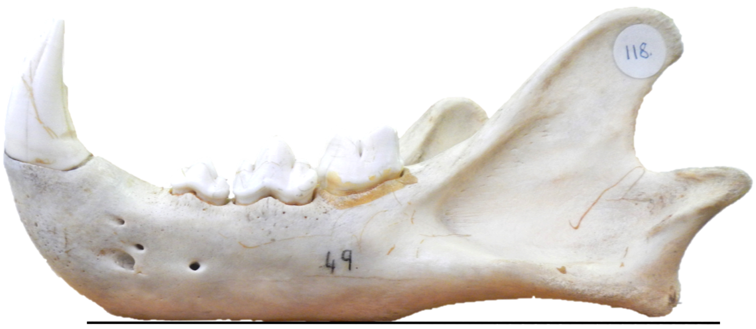

Part D S2 Fig. Non-rocking lion mandible (WITS 49). Specimen of unknown sex and origin. The ventral margin of the horizontal ramus is almost flat. *[Photo: V.L. Williams]*

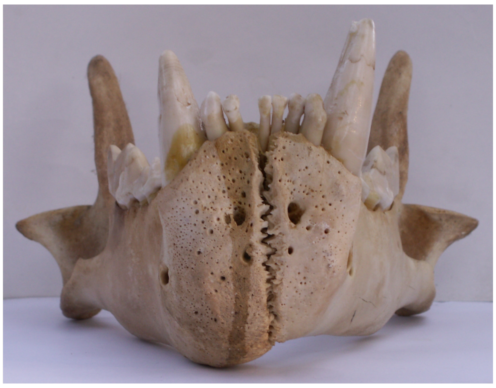

Part E S2 Fig. Non-rocking mandible of a 3 year-old male lion from Hwange, Zimbabwe (SPIcM6PAC). The mandible is distorted by a bony growth on one side resulting from a tooth infection. *[Photo: J. Hunt]*

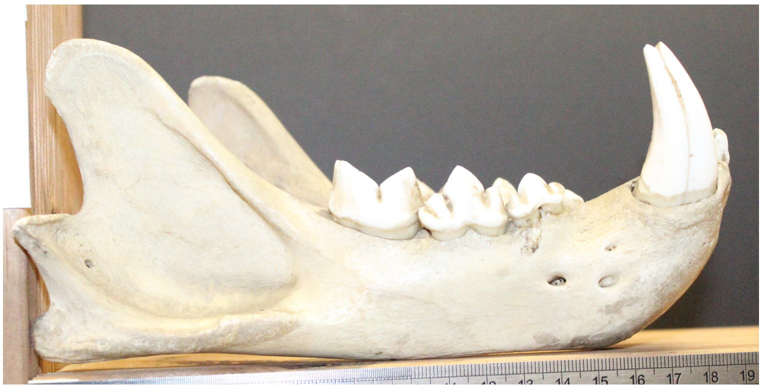

Part F S2 Fig. Rocking tigress mandible (OUMNH 14751). *[Photo: A.J. Loveridge]*
