# Supplementary material for: ‘Skullduggery’: Lions Align and Their Mandibles Rock!
Source: PLoS One. 2015 Nov 4;10(11):e0135144. doi: 10.1371/journal.pone.0135144 (PMC4633142; doi:10.1371/journal.pone.0135144)
Supplement: S3 Fig — (PDF) [file pone.0135144.s003.pdf]

S3 Figures. Features of cranial morphology in lions.

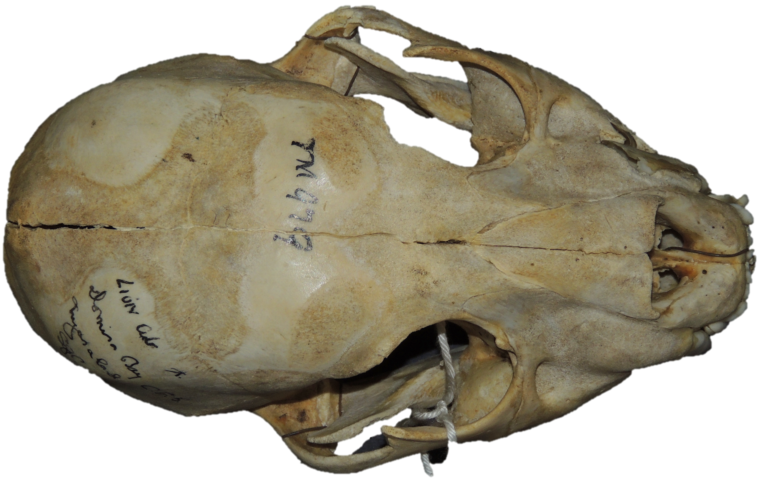

Part A S3 Fig. Juvenile lion cranium (DMNH, TM 979) showing normal alignment of maxilla-nasal-frontal sutures. Specimen from Tanzania. *[Photo: V.L. Williams]*

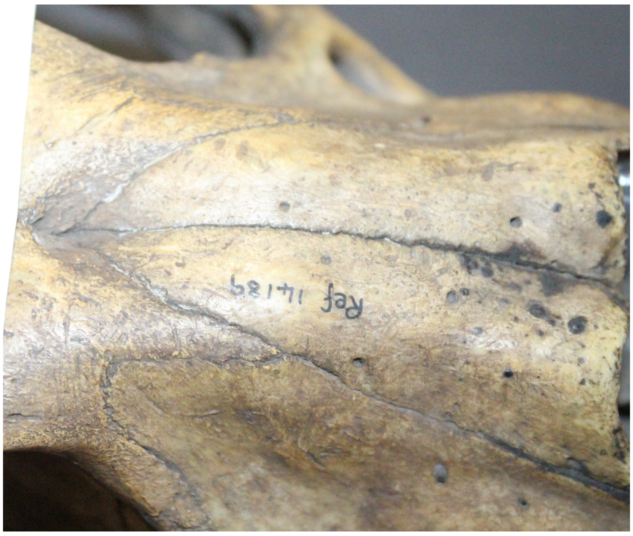

Part B S3 Fig. Lioness cranium from Sudan (OUMNH 14189). Apex of the nasal-frontal sutures slightly posterior to the apices of the maxilla-frontal sutures. *[Photo: A.J. Loveridge]*

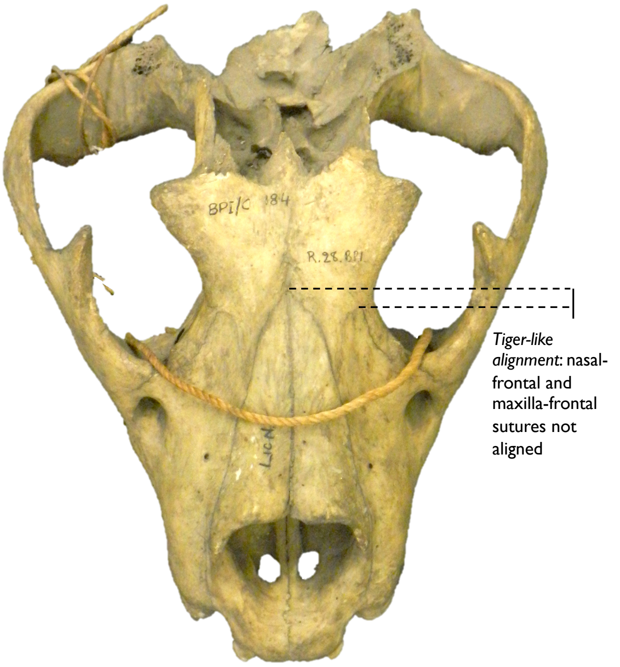

Part C S3 Fig. Cranium of a lion of unknown sex and origin (EIS BP/4/184). Nasal-frontal sutures extend more posterior than the maxilla-frontal sutures. *[Photo: V.L. Williams]*

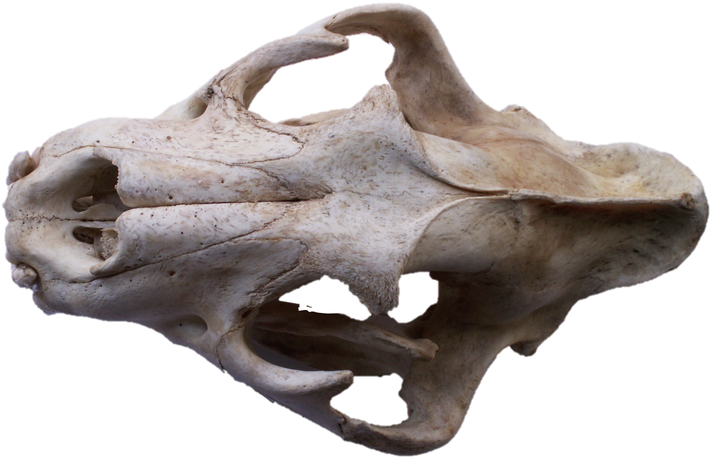

Part D S3 Fig. Cranium of 7.5-year-old lion from Hwange (NEHcM4). The apices of the nasal-frontal sutures are very slightly anterior to the apices of the maxilla-frontal sutures and are not aligned. *[Photo: J. Hunt]*
