# Supplementary figures and images for: ‘Skullduggery’: Lions Align and Their Mandibles Rock!
Source: PLoS One. 2015 Nov 4;10(11):e0135144. doi: 10.1371/journal.pone.0135144 (PMC4633142; doi:10.1371/journal.pone.0135144)

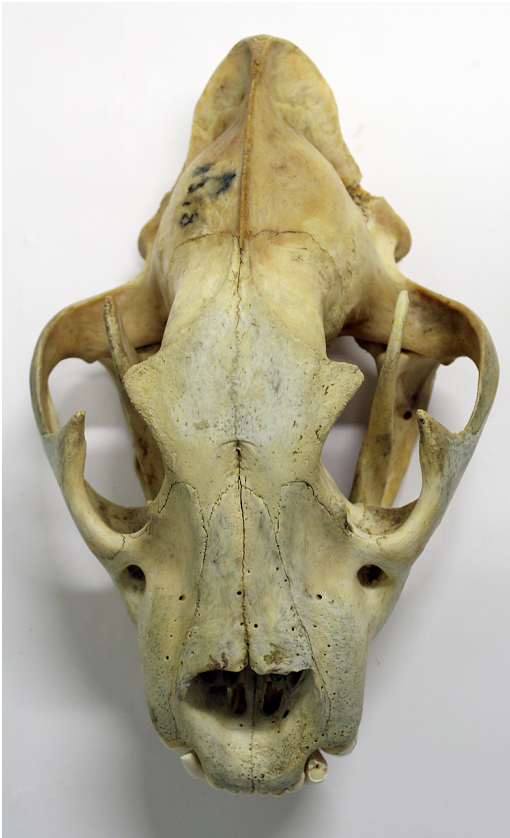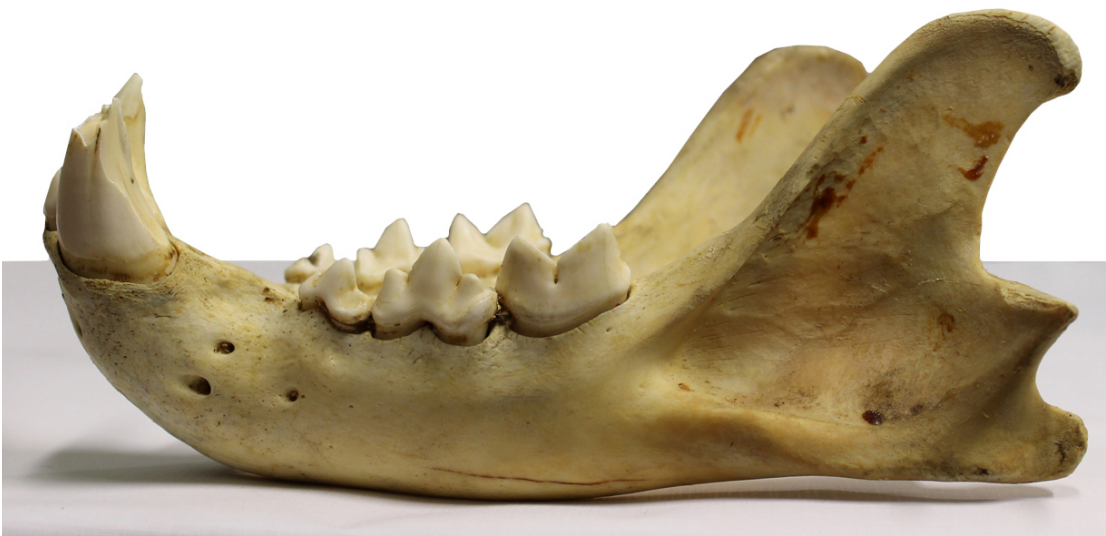

**S4 Fig. 'Liger' cranium and mandible (*P. leo* ♂ x *P. tigris* ♀) (NMB 934) [Photos: National Museum, Bloemfontein]**

Supplement: S4 Fig — (PDF) [file pone.0135144.s004.pdf]
