# Supplementary material for: ‘Skullduggery’: Lions Align and Their Mandibles Rock!
Source: PLoS One. 2015 Nov 4;10(11):e0135144. doi: 10.1371/journal.pone.0135144 (PMC4633142; doi:10.1371/journal.pone.0135144)
Supplement: S1 Table — (PDF) [file pone.0135144.s005.pdf]

S1 Tables. Accession numbers and information for African Lion (*Panthera leo*) skull and skeleton specimens

Part A S1 Table. Skull specimens from the Ditsong Museum of Natural History (DMNH), Pretoria, South Africa (formerly the Transvaal Museum, TM). *[Abbreviations: M=Male, F=Female; ?=unknown; N=No, Y=Yes, I=Intermediate]*

Accession Number	Skull Mass (kg) (cranium & mandible)	Sex	Mortality Date	Country of Origin	Cranial sutures align?	Mandibles rock?
385	0.75	F	December 1906	South Africa	Y	Y
AZ2389	1.77	M	?	?	Y	Y
AZ566	1.84	M	?	?	Y	Y
AZ771	1.36	F	?	?	Y	Y
AZ947	?	F	?	?	Y	Y
CR9	1.10	?	1894	Zimbabwe	Y	? [a]
TM765	1.27	F	1932	South Africa	Y	? [a]
TM868	0.93	F	?	South Africa	Y	? [a]
TM869	1.80	M	?	South Africa	Y	Y
TM926	1.12	F	September 1928	South Africa	Y	Y
TM927	1.80	M	1908	Mozambique	Y	Y
TM964	1.21	M	1937	South Africa	Y	? [b]
TM973	2.15	?	before 1925	?	Y	I [c]
TM978	1.79	M	August 1943	Malawi	Y	N
TM1023	1.76	M	February 1945	Namibia	Y	I [c]
TM1024	1.89	M	1945	Namibia	Y	? [d]
TM1026	1.20	F	?	South Africa	Y	I [c]
TM3186	0.90	M	?	Sudan	Y	? [a]
TM3187	1.17	F	?	Sudan	Y	? [a]
TM3187	0.82	F	?	Sudan	Y	Y
TM4403	1.30	F	February 1925	South Africa	Y	? [d]
TM5604	0.83	M	November 1928	South Africa	Y	Y
TM12428	1.31	M	?	Namibia	Y	Y
TM13924	0.76	?	?	South Africa	Y	Y

TM16723	0.72	?	?	?	Y	Y
TM16736	1.22	?	?	?	Y	? [b]
TM24004	1.20	M	May 1974	South Africa	Y	Y
TM38242	2.03	?	?	South Africa	Y	I [c]
TM38243	1.25	F	April 1969	Namibia	Y	? [d]
TM38244	1.64	F	?	South Africa	Y	? [d]
TM38245	1.09	F	?	Namibia	Y	I [c]
TM38246	1.27	M	1974	South Africa	Y	N (see Part A in S2 Fig.)
TM38247	?	M	1968	Namibia	Y	? [d]
TM38248	0.94	M	September 1957	Namibia	Y	? [a]
TM38249	1.93	M	?	South Africa	Y	Y
TM38250	1.94	F	September 1964	Namibia	Y	? [d]
TM38251	1.98	M	?	?	Y	Y
TM38252	1.15	F	?	South Africa	Y	Y
TM38253	0.88	F	1974	South Africa	Y	N (see Part B in S2 Fig.)
TM38255	1.02	?	?	Namibia	Y	Y
TM979	? - juvenile	?	September 1941	Tanzania	Y (see Part A in S3 Fig.)	Y

[a] Could not establish because the jaw was wired shut;
[b] Incomplete mandible;
[c] Exhibition of this trait was intermediate (I). One dentary had one contact point below the carnassials and rocked, whereas the other dentary had an additional contact point on the angular process and therefore touched a flat surface at two points.
[d] No mandible

Part B S1 Table. Skull specimens from the Oxford University Museum of Natural History (OMONH) *[Abbreviations: M=Male, F=Female; ?=unknown; N=No, Y=Yes, I=Intermediate]*

Accession Number	Skull Mass (kg) (cranium & mandible)	Sex	Mortality Date	Country of Origin	Cranial sutures align?	Mandibles rock?
9172	1.17	F	Before 1957	Tanzania	Y	Y [a]
14173	1.02	F	Before 1860	?	Y	Y [a]
14174	1.00	F	Before 1876	India	Y	Y [a]
14175	1.99	M	Before 1860	?	Y	Y
14178	1.03	F	Before 1865	South Africa	Y	Y
14179	1.57	M	Before 1865	South Africa	Y	Y
14181	1.19	F	Before 1860	South Africa	Y	Y
14182	?	F	1902-1912	Sudan	Y	Y [a,b]
14183	1.03	F	1906	Sudan	Y	Y [a,b]
14184	0.85	F	1907	Sudan	Y	N [a,b] (see Part C in S2 Fig.)
14185	?	F	1902-1912	Sudan	Y	Y [b]
14186	0.88	F	1902-1912	Sudan	Y	Y [a,b]
14187	0.86	F	1902-1912	Sudan	Y	Y [b]
14188	0.79	F	1902-1912	Sudan	Y	Y [b]
14189	0.91	F	May 1905	Sudan	N (see Part B in S3 Fig.)	Y
14190	?	M	May 1905	Sudan	Y	Y [a]
14192	0.95	F	1906	Sudan	Y	Y
14193	0.89	F	1906	Sudan	Y	Y
14194	1.13	F	December 1872	Uganda	Y	Y
14196	1.82	M	Before 1937	Uganda / Sudan	Y	Y
14200	1.61	M	?	?	Y	Y
14202	0.84	?	?	?	Y	Y
14204	1.37	F	Before 1860	?	Y	Y
1830?	?	M	?	?	Y	Y [a]
14191	0.55 - juvenile	F	1902-1912	Sudan	Y	Y

[a] Bony spur below the mandibular symphysis;
[b] convexity of the horizontal ramus is less pronounced and less rounded and is rather ‘flatish’ (e.g. Part C in S2 Fig.)

Part C S1 Table. Skull specimens from the School of Animal, Plant & Environmental Sciences (WITS or WLSM), University of the Witwatersrand, South Africa. *[Abbreviations: ?=unknown; N=No, Y=Yes]*

**Accession Number**	**Skull Mass (kg) (cranium & mandible)**	**Sex**	**Mortality Date**	**Country of Origin**	**Cranial sutures align?**	**Mandibles rock?**
49	1.617	?	?	?	Y	N [a] (see Part D in S2 Fig.)
55	0.948	?	?	?	Y	Y
1448	1.160	?	1993	Probably South Africa	Y	Y

[a] The horizontal ramus was almost straight from the mandibular symphysis to the region below the last molar. The angular process did not touch the flat surface.

Part D S1 Table. Skull specimens from the Evolutionary Studies Institute (ESI), University of the Witwatersrand, South Africa. *[Abbreviations: M=Male, ?=unknown; N=No, Y=Yes, I=Intermediate]*

**Accession Number**	**Skull Mass (kg) (cranium & mandible)**	**Sex**	**Mortality Date**	**Country of Origin**	**Cranial sutures align?**	**Mandibles rock?**
BP/4/183	1.162	?	?	?	Y	Y
BP/4/184	?	?	?	?	N (see Part C in S3 Fig.)	I
BP/4/186	?	M	?	South Africa	Y	? [a]
BP/4/187	0.930	?	?	?	Y	Y
Za 17	1.845	?	March 1952	?	Y	I

[a] No mandible

Part E S1 Table. Skull specimens from Hwange National Park, Zimbabwe. The skulls are the property of the Zimbabwe Parks & Wildlife Management Authority at Main Camp, Hwange National Park, Zimbabwe and are not housed or curated properly. The skulls were given a study ID number by the Hwange Lion project of WildCRU (Oxford University). Access to the skulls can be obtained by contacting Jane Hunt. *[Abbreviations: M=Male, F=Female; ?=unknown; N=No, Y=Yes, I=Intermediate]*

**Study ID Number**	**Skull Mass (kg) (cranium & mandible)**	**Sex**	**Known age**	**Mortality Date**	**Country of Origin**	**Cranial sutures align?**	**Mandibles rock?**
SPIcM6 PAC	1.925	M	3 years	December 2010	Zimbabwe	Y	I [c] (see Part E in S2 Fig.)
LFMM1	2.200	M	7 years	December 2012	Zimbabwe	Y	I [d]
DEKM1	?	M	7 years	December 2013	Zimbabwe	Y	Y
NEHcM4	2.675	M	7.5 years	May 2013	Zimbabwe	N [b] (see Part D in S3 Fig.)	Y
SOAdF1	1.400	F	5 years	June 2013	Zimbabwe	Y	Y [e]
CATF2	1.470	F	7.5 years	May 2012	Zimbabwe	Y	Y
No number [a]	1.570	F	Est. 5 years	May 2011	Zimbabwe	Y	Y
NEHbF1	1.650	F	10 years	June 2013	Zimbabwe	Y	Y [f]

[a] Unknown female; poached; no study number

[b] Posterior projections of nasal-frontal sutures very slightly anterior to the apex of the maxilla-frontal sutures

[c] The front of one dentary was calloused due to infection in the teeth, hence the mandible was distorted

[d] Exhibition of this trait was intermediate (I). One dentary had one contact point below the carnassials and rocked, whereas the other dentary had an additional contact point on the angular process and therefore touched a flat surface at two points.

[e] Had a tendency to rock forward because of disease/calloused infection in the bone that added extra bone to the front of the mandible

f Had a tendency to rock forward because of bony spur below the mandibular symphysis.

Part F S1 Table. Skeleton specimens from the Ditsong Museum of Natural History (DMNH), Pretoria, South Africa

Accession Number	Skeleton Mass (kg)	Skull present (P) or absent (A)	Sex	Country of Origin
AZ565	6.25	A	M	?
AZ2389	10.76 (post-cranial only = 8.99)	P	M	?

Part G S1 Table. Skeleton specimens from 15 consignments of skeletons.

Consignment mass (kg)	Number of skeletons per consignment	Skull present (P) or absent (A)	Sex	Country of Origin
47.6	5	?	?	South Africa
76.0	10	?	?	South Africa
129.0	12	?	?	South Africa
146.0	13	?	?	South Africa
137.6	14	?	?	South Africa
144.0	19	?	?	South Africa
192.0	19	?	?	South Africa
247.0	25	?	?	South Africa
366.0	35	?	?	South Africa
364.0	39	?	?	South Africa
380.0	43	?	?	South Africa
409.0	46	?	?	South Africa
661.0	64 [a]	?	?	South Africa
651.0	66	?	?	South Africa
947.0	100 [a]	?	?	South Africa

[a] data corrected for anomalies; original values were half the quantity listed above.

Part H S1 Table. Skeleton specimens from 34 bags of individual skeletons.

Skeleton Mass (kg)	Skull present (P) or absent (A)	Sex	Country of Origin
6.5	P	?	South Africa
7.0	P	?	South Africa
7.5	P	?	South Africa
8.0	P	?	South Africa
8.0	P	?	South Africa
8.5	P	?	South Africa
8.5	P	?	South Africa
10.5	P	?	South Africa
13.0	P	?	South Africa
13.0	P	?	South Africa
6.0	A	?	South Africa
6.0	A	?	South Africa
6.0	A	?	South Africa
7.0	A	?	South Africa
7.0	A	?	South Africa
7.0	A	?	South Africa
7.0	A	?	South Africa
7.0	A	?	South Africa
8.0	A	?	South Africa
8.0	A	?	South Africa
6.7	?	?	South Africa
7.2	?	?	South Africa
8.7	?	?	South Africa
9.1	?	?	South Africa
9.6	?	?	South Africa
10.1	?	?	South Africa
10.5	?	?	South Africa

10.6	?	?	South Africa
10.6	?	?	South Africa
10.6	?	?	South Africa
10.7	?	?	South Africa
10.7	?	?	South Africa
11.1	?	?	South Africa
11.4	?	?	South Africa
