# Supplementary material for: ‘Skullduggery’: Lions Align and Their Mandibles Rock!
Source: PLoS One. 2015 Nov 4;10(11):e0135144. doi: 10.1371/journal.pone.0135144 (PMC4633142; doi:10.1371/journal.pone.0135144)
Supplement: S2 Table — (PDF) [file pone.0135144.s006.pdf]

S2 Tables. Accession numbers and information for tiger (*Panthera tigris* subsp.) skull specimens

Part A S2 Table. Specimens from the Oxford University Museum of Natural History (OUMNH). *[Abbreviations: M=Male, F=Female; ?=unknown; N=No, Y=Yes]*

Accession Number	Sex	Mortality Date	Country of Origin	Cranial sutures align?	Mandibles rock?
2980	M	Before 1879	?	N	N
8995	M	1871	?	N	N
14195	M	Before 1937	?	N	N
14212	M	Before 1860	?	N	N
14213	F	Before 1860	?	N	N
14214	F	Before 1860	?	N	N
14215	M	1854	?	N	N
14216	M	Before 1860	?	N	N
14217	F	?	?	N [a]	N
14220	M	Before 1930	India	N	N
14221	M	Before 1915	'Bengal'	N	N
14222	F	June 1831	India	N	N
14223	?	November 1885	Malaysia	N	N
14226	?	?	?	N	N
14227	M	?	?	N	N
14228	M	?	?	N [b]	N
14751	F	Before 1860	?	N	Y [c] (see Part F in S2 Fig.)
14752	?	Before 1878	?	? [d]	N
17379	F	?	India	N	? [e]

[a] very close to alignment, skull bisected;
[b] nasals much shorter than other specimens examined;
[c] jaw rocks very slightly;
[d] no cranium;
[e] mandible damaged.

Part B S2 Table. Specimens from the Ditsong Museum of Natural History (DMNH), Pretoria, South Africa (formerly the Transvaal Museum, TM). *[Abbreviations: M=Male, F=Female; ?=unknown; N=No]*

Accession Number	Sex	Mortality Date	Country of Origin	Cranial sutures align?	Mandibles rock?
AZ772	F	?	?	N	N
AZ1045	M	?	?	N	N
TM157	?	?	China	N	N
